# Effects of Long-Term Feeding of the Polyphenols Resveratrol and Kaempferol in Obese Mice

**DOI:** 10.1371/journal.pone.0112825

**Published:** 2014-11-11

**Authors:** Mayte Montero, Sergio de la Fuente, Rosalba I. Fonteriz, Alfredo Moreno, Javier Alvarez

**Affiliations:** Instituto de Biología y Genética Molecular (IBGM), Departamento de Bioquímica y Biología Molecular y Fisiología, Facultad de Medicina, Universidad de Valladolid and Consejo Superior de Investigaciones Científicas (CSIC), Valladolid, Spain; Texas Tech University Health Science Centers, United States of America

## Abstract

The effect of the intake of antioxidant polyphenols such as resveratrol and others on survival and different parameters of life quality has been a matter of debate in the last years. We have studied here the effects of the polyphenols resveratrol and kaempferol added to the diet in a murine model undergoing long-term hypercaloric diet. Using 50 mice for each condition, we have monitored weight, survival, biochemical parameters such as blood glucose, insulin, cholesterol, triglycerides and aspartate aminotransferase, neuromuscular coordination measured with the rotarod test and morphological aspect of stained sections of liver and heart histological samples. Our data show that mice fed since they are 3-months-old with hypercaloric diet supplemented with any of these polyphenols reduced their weight by about 5–7% with respect to the controls fed only with hypercaloric diet. We also observed that mice fed with any of the polyphenols had reduced levels of glucose, insulin and cholesterol, and better marks in the rotarod test, but only after 1 year of treatment, that is, during senescence. No effect was observed in the rest of the parameters studied. Furthermore, although treatment with hypercaloric diets induced large changes in the pattern of gene expression in liver, we found no significant changes in gene expression induced by the presence of any of the polyphenols. Thus, our data indicate that addition of resveratrol or kaempferol to mice food produces an initial decrease in weight in mice subjected to hypercaloric diet, but beneficial effects in other parameters such as blood glucose, insulin and cholesterol, and neuromuscular coordination, only appear after prolonged treatments.

## Introduction

The importance of the presence of vegetables in human food has been widely debated, and in the last years considerable scientific literature has been published in relation with the effects in health of diets rich in vegetables or in particular types of polyphenols. Most of these studies are epidemiological, and associate the amount of polyphenols present in the diet with a reduction in the risk for cardiovascular diseases and cancer [1]–[8]. Some studies have also described other positive effects in neurodegenerative diseases [9], [10] or inflammatory diseases [11], and they have also been shown to activate lipolysis [12], [13]. All these potentially beneficial effects have led to proposals for their addition in foods as a nutritional supplement.

Moreover, some studies have investigated the effects of some of these individual molecules in cellular or animal models. The more striking example is resveratrol, a polyphenol abundant in red wine, which has been shown to improve the survival and several plasmatic, neurological and genomic markers in mice fed with hypercaloric diets [14]. Previously, it had been shown that this compound was able to prolong considerably the lifespan in several species, including Saccharomyces cerevisiae, Caenorhabditis elegans, Drosophila melanogaster and a short-lived fish [15]–[19]. More recently, it has also been shown to protect pancreatic islets against oxidative stress in db/db mice [20]. These effects have been attributed not only to the antioxidant properties of this compound, but rather to the specific activation by resveratrol of sirtuins, a family of NAD^+^-dependent deacetylases and mono ADP-rybosiltransferases that mediate the physiological effects of caloric restriction. From these data an entire industry has emerged to provide food supplements, cosmetics, and other products based on resveratrol. However, recent data has questioned the results obtained with resveratrol in C. elegans and D. melanogaster [21] and this point is now a matter of strong debate [22]–[24].

Although at a smaller scale, similar studies have also been carried out with other related polyphenols, but significant differences appear among the reported effects. We were particularly interested in the flavonoid kaempferol, as we showed several years ago that this compound was a potent stimulator of the mitochondrial Ca^2+^ uniporter, a Ca^2+^ channel essential to modulate mitochondrial energy production [25]. It has been shown that kaempferol increases oxygen consumption in cell cultures and isolated mitochondria, potently activates the synthesis of thyroid hormones and modulates a whole series of enzymes and proteins related to metabolism [26]. In addition, a 30 days administration of a kaempferol glucoside to Wistar rats feed with hypercaloric diet reduced the weight and the plasmatic levels of triglycerides [27]. Kaempferol, however, was much less effective as activator of sirtuins [15], and resveratrol, instead, was ineffective as stimulator of thyroid hormone synthesis [14] and little effective as activator of mitochondrial Ca^2+^ uptake [25].

Both compounds (resveratrol and kaempferol) thus seem to act through different signaling pathways, and we thought it would be very interesting to compare their effects on several biochemical, neurological and genomic parameters during a lifetime treatment with these compounds in mice. We have then decided to make a large and complete study of this point by using a very large number (250) 3-months old C57BL mice and carrying out the treatment for the entire life of the mice (24 months). As in previous reports [14], [27], we have also studied the effects of these polyphenols in mice fed with hypercaloric diet, periodically monitoring parameters such as food intake, weight, blood glucose, insulin, triglycerides, cholesterol and transaminase activity. Motor coordination was estimated using the rotarod test [28], [29], and we have also compared mortality rates, analyzed histological sections of liver and heart, and looked for changes in gene expression patterns in liver using Affymetrix gene expression arrays.

## Methods and Materials

### Ethics statement

The study was approved by the Ethical Committee for Animal Research of the School of Medicine of the University of Valladolid (Permit Number: 7-2007), and was carried out in strict accordance with the recommendations in the Guide for the Care and Use of Laboratory Animals of the National Institutes of Health.

### Mice treatment and diets

250 C57BL male mice were obtained from Janvier (53947 Saint Berthevin Cedex, France) and fed with control diet (Diet D12450B from Research Diets Inc., New Brunswick, NJ 08901 USA) until they were 3-months old and at that moment the different diets were started. This diet contains 3.85 kcal/g. Mice were maintained in standard housing conditions at 22°C, with a 12 h light/dark cycle and with free access to tap water. They were distributed in 5 groups with 50 mice in each of them, and placed in cages containing 10 mice in each for all their life. Group Control (C) continued with the same control diet. Group Hypercaloric Control (HC) was fed with a standard hypercaloric diet (Diet D12492 from Research Diets Inc.), a diet containing a larger proportion of lard and providing 5.24 kcal/g. Control diet contains 1.9% lard, providing 4.44% of the total kcal, while hypercaloric diet contains 31.66% lard, providing 54.35% of the total kcal. Group Hypercaloric Resveratrol (HR) was fed with hypercaloric diet (D12492) supplemented with 250 mg/kg-diet (0,025%) of resveratrol. Group Hypercaloric low Kaempferol (Hk) was fed with hypercaloric diet (D12492) supplemented with 50 mg/kg-diet (0,005%) of kaempferol. Group Hypercaloric high Kaempferol (HK) was fed with hypercaloric diet (D12492) supplemented with 250 mg/kg-diet (0,025%) of kaempferol. Food was stored at 4°C, sealed and at dark. Both resveratrol and kaempferol were obtained from Apin Chemicals Limited, Abingdon, Oxon, OX14 4RU, U.K., and sent to Research Diets to prepare the different types of foods, which were identified by color codes.

**Figure 1:**
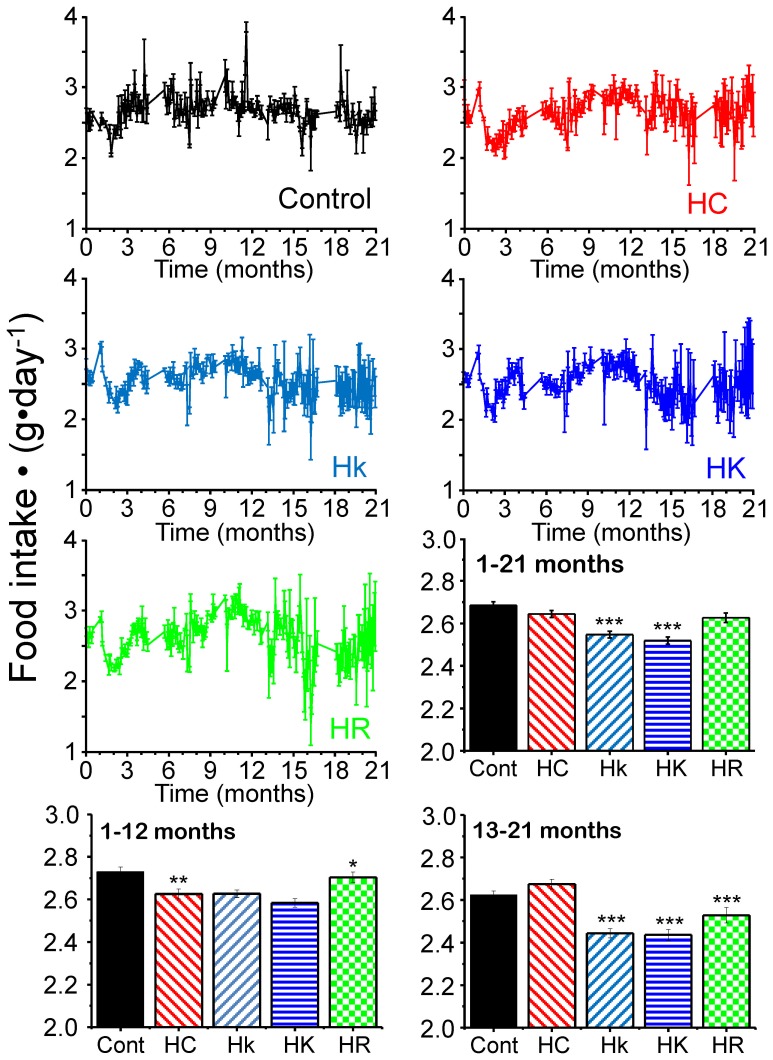
Variation of the mean amount of food ingested by each group of mice along their lifespan. Data are calculated as food intake per mice and per day. Each data was obtained as mean±s.e, n = 5 (5 mice cages containing 10 mice each). The bar diagrams shows the mean±s.e. of all the measurements obtained for each group of mice (n = 197 measurements for whole period, n = 112 for the first 12 months, n = 85 for 13–21 months) and the significance (*, p<0.05; **, p<0.005; ***, p<0.001) of the differences (HC group, compared with the control; Hk, HK and HR groups, compared with the HC group).

**Figure 2:**
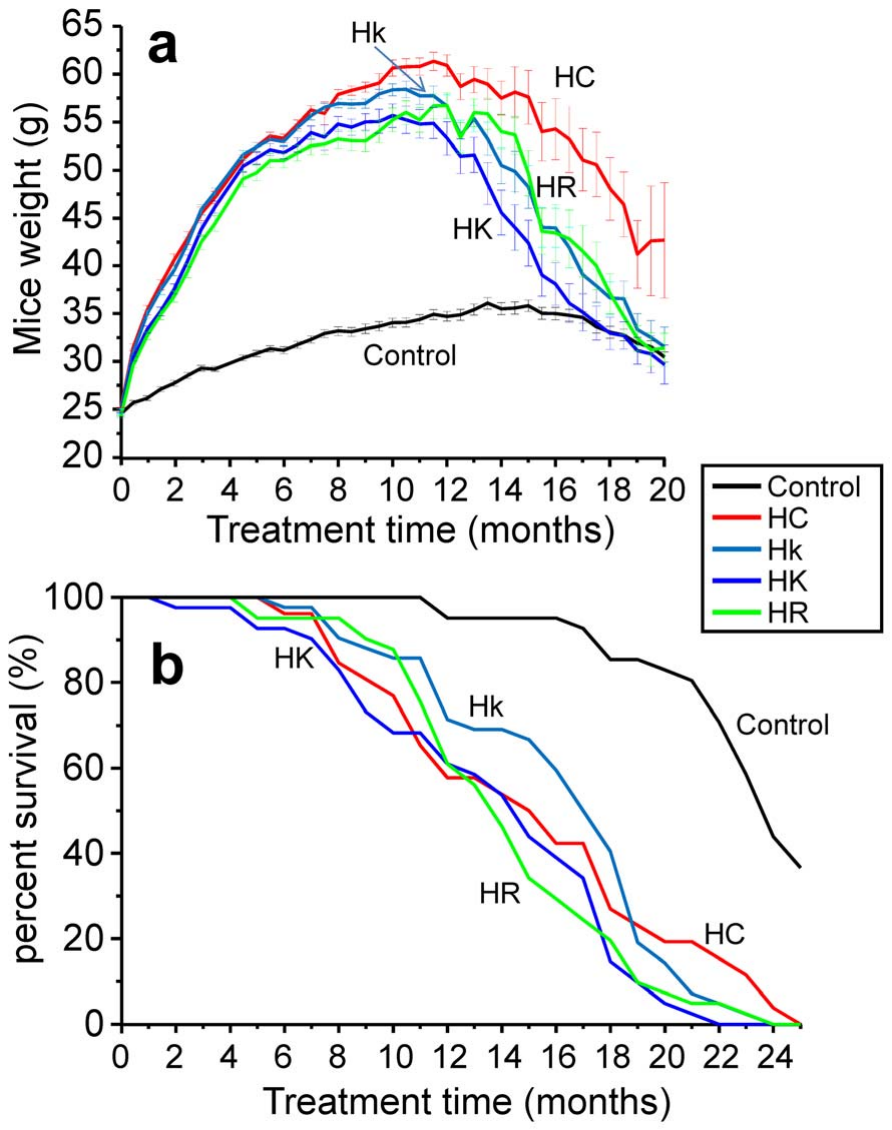
Evolution with time of mice weight and survival. **Panel a**. Evolution of mice weight along the study from the start of the hypercaloric diets (time 0 in the figure). Data are mean±s.e, n = 50 data for each value at the beginning, the number of data then decreasing with time according to the survival. **Panel b**. Evolution of mice survival with time in all the groups, expressed as percentage of the starting mice number.

The amount of food ingested was measured by putting known amounts of food in every cage and weighting the remaining food every two days. As shown in Fig. 1 and Table S1, the amount of food ingested kept quite constant along the whole study for all the conditions, although some small changes among the groups could be detected (see Results). The initial weight of the mice was around 25 g, and so the initial dose of the compounds was 25 mg⋅kg^−1^⋅day^−1^ in groups HR (as in ref. 14) and HK, and 5 mg⋅kg^−1^⋅day^−1^ in group Hk. Mice fed hypercaloric diets increased progressively weight along the study (Fig. 2a), reaching values of 55–60 g after 1 year of treatment.

Once the treatment was started, we monitored a series of parameters along the whole life of the mice: mice weight every 15 days, blood glucose, insulin, triglycerides, cholesterol and aspartate aminotransferase every three months, neurological performance with the rotarod test every 3 months, histological studies of liver and heart after 6 and 12 months of hypercaloric diet, and gene expression studies with Affymetrix arrays in liver extracts after 12 months.

### Blood biochemistry

Glucose was measured using the Ascensia Breeze 2 Blood Glucose Meter (Bayer HealthCare AG, Wuppertal, Germany). Insulin was measured by ELISA using the kit for mouse insulin from DRG Diagnostics, Marburg, Germany (Ref. EIA-3439). Triglycerides, total cholesterol and alanine aminotransferase were measured using the corresponding kits from BioSystems, Barcelona, Spain (Refs. 11528, 11505 and 11533, respectively). All these measurements were made using blood taken from the tail after overnight fast.

### Rotarod test

The Ugo Basile (Comerio, Italy) Mouse Rota Rod was used for the tests. Starting at 0 rpm, the rotarod was accelerated at constant rates of 4–40 rpm. All the mice in the same cage were first trained on the rotarod the day before at a constant rate of 4 rpm until they were able to stay on for one minute. The next day, 5 mice were randomly chosen in that cage and labeled. Then each mice was subjected to three rotarod trials, with 30 min intervals, consisting in a constant acceleration along 5 min from 4 rpm to 40 rpm. The final time to fall of each mouse was obtained as the mean±s.e. of all the measurements for that mouse. Rotarod tests were carried out every 3 months in all the mice groups.

### Histological studies

Mice were sacrificed by cervical dislocation. The heart and liver were removed and washed several times in 4°C cold PBS. Tissues were cut in small pieces and fixed for 24 hours in 4% formalin at 4°C. After fixation, tissues were washed 3 times for 30 min in cold PBS, once in distilled water and finally once in 70% ethanol. The tissue sections were then embedded in paraffin in a carousel tissue processor cut in 4 µm sections and mounted on Superfrost slides. They were then let dry overnight at 37°C before they were finally stained with Haematoxyline and Eosin and imaged in a Nikon Eclipse 80i upright microscope at 20× and 40×.

### Gene expression

Three mice of each group (3×5) were sacrificed and their livers were separately minced, immersed in RNAlater RNA stabilization Reagent (Quiagen) and frozen. For RNA extraction, liver chunks were homogenized using the Minibeadbeater-8, and RNA was then extracted using the RNeasy mini kit (Qiagen). RNA concentrations were measured using a NanoDrop spectrophotometer (NanoDrop Technologies, Wilmington, DE, USA), and samples were analyzed using 15 Mouse Gene 1.0 ST arrays, one for each mice, in the Genomic facility of the Cancer Research Center, Salamanca, Spain. Background correction, intra- and inter-microarray normalization, and expression signal calculation was made using a robust microarray analysis algorithm [30]–[32]. This algorithm calculates the absolute expression signal for each gene in each microarray. Then, a method known as significance analysis of microarray [33] was applied to calculate significant differential expression. This method provides an estimation of the error using the false discovery rate algorithm (FDR) [34]. A FDR of less than 0.1 (10% false positives) was used as standard for all the differential expression calculations, although in some cases it was increased to 0.15 (15% false positives), as indicated. All these methods were applied using R and Bioconductor packages.

### Statistics

Data are expressed as mean±s.e. Significance analysis was made with the ANOVA test.

## Results

### Food ingestion

The mean amount ingested by the group treated with HC diet was significantly smaller in the first 12 months, although its caloric value was still much higher (see Fig. 1 and Table S1). Food intake was little modified in the groups treated with the drugs compared with the HC group in the first 12 months, but it was significantly decreased (by 5–9%) in the senescence period (13–21 months).

### Mice weight

Control mice (C) started with a weight of about 25 g, which increased to stabilize at around 30–35 g at one year, and then slowly declined with senescence (see figure 2a). The weight of mice fed high-calorie diet (HC) increased rapidly, reaching around 60 g after one year of treatment with this diet. Then it started to decrease with senescence, tending to return to the original values after 2 years of treatment. Mice treated with HC diet with resveratrol (HR, 25 mg/kg body weight per day) increased weight 7.0±0.4% less (mean±s.e. obtained from the first 20 weight measurements, obtained every 15 days) than HC mice during the first year, and this effect was statistically significant for every measurement (see Table S2). Then, weight also began a rapid decline with senescence, in parallel with the weight loss observed in the HC mice, but starting about 3 months earlier. In the case of mice treated with kaempferol at low concentration (Hk, 5 mg/kg body weight per day), there were no significant differences in weight during most of the first year of treatment (Table S2), but the subsequent drop in weight associated with senescence also occurred about 3 months earlier than in the case of the controls with HC diet. In the case of mice treated with HC diet and kaempferol at high concentration (HK, 25 mg/kg body weight per day), there was also an initial decrease in weight of a 5.0±0.5% (mean±s.e. obtained from the first 20 weight measurements, obtained every 15 days), which was statistically significant for most of the measurements, but not all of them (Table S2). Then, the subsequent decline in weight associated with senescence began even earlier, around 4 months earlier than in control mice treated with HC diet, so that the differences in weight with the HC group remained highly significant for most of the senescence period (Table S2). In summary, kaempferol and resveratrol treatments produced significant decreases (5–7%) in weight in mice fed with HC diet during the first year of treatment, and induced an early start of the decrease in weight associated with senescence.

### Survival study

In control mice, mortality was very small before they were 18 months old. Feeding with HC diet significantly reduced survival (Fig. 2b), so that 50% mortality was already reached by 15 months of life (compared with only 5% mortality in controls of the same age). The administration of dietary kaempferol or resveratrol at high concentrations did not significantly change the mortality curve with respect to that of the HC mice. In the case of kaempferol at low concentration, 50% mortality was obtained 2 months later, at 17 months, but the subsequent decrease of the survival curve was faster, equaling with the group of mice with HC diet at 19 months. In summary, our data show that treatment with kaempferol or resveratrol did not modify survival in mice fed with hypercaloric diet.

### Glucose

**Figure 3:**
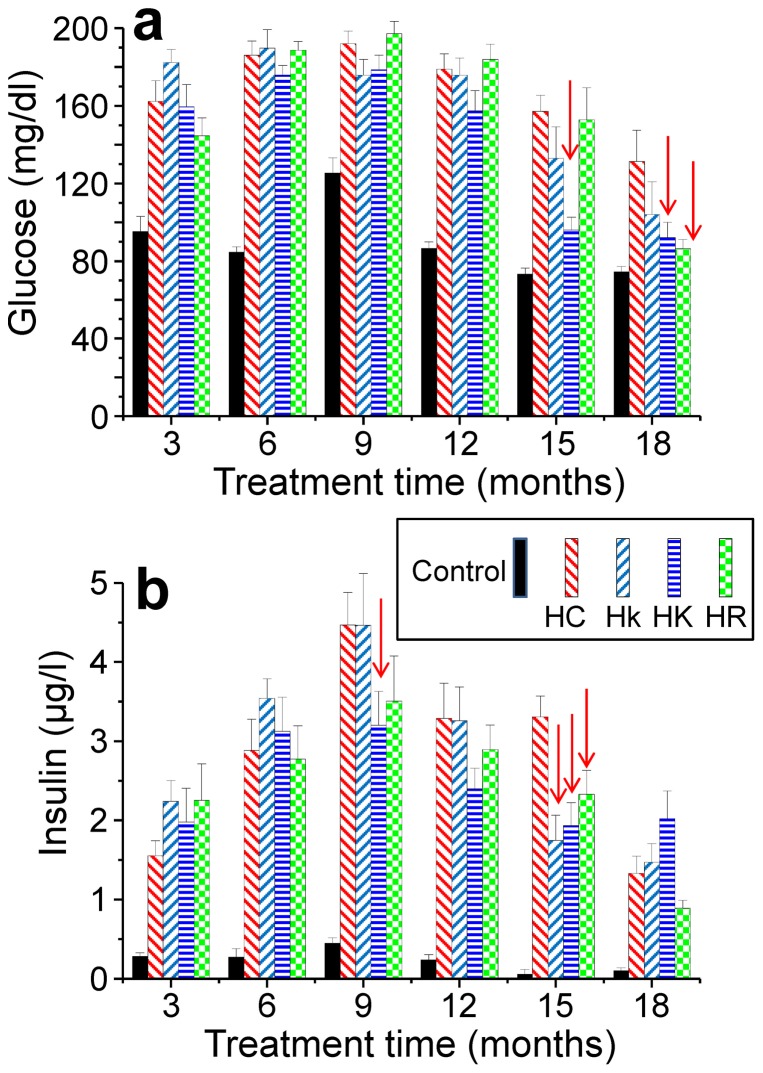
Mean blood glucose (panel a) and insulin (panel b) values obtained every 3 months along the study, from the start of the hypercaloric diet. Arrows indicate the presence of differences statistically significant when the HC condition is compared to the Hk, HK and HR ones (see the text). Data are mean±s.e, n = 10 data for each value.

Blood glucose levels in control mice were stable around 80–120 mg/dl throughout all the study. In all the other conditions having HC diet, there was a rapid increase in the blood glucose levels, which doubled the previous glucose levels immediately after establishment of the HC diet treatment (Fig. 3a). These high levels were not significantly modified by any of the polyphenols used throughout the first year of treatment. Then, during senescence, there was a slight decrease in the glucose levels, which was more rapid and pronounced in mice treated with polyphenols. In particular, kaempferol at high doses produced a significant decrease with respect to the HC mice (p<0.005, ANOVA test) in blood glucose levels at 15 months of treatment (see red arrows), and both kaempferol and resveratrol produced a decrease (p<0.05) in these values at 18 months. Therefore, significant effects of the polyphenols were only observed after more than 1 year of treatment.

### Insulin

Insulin levels in control mice remained around 0.2 to 0.3 µg/l throughout the study. In contrast, in all conditions of HC diet-fed mice there was a large increase in blood insulin levels to values 10–15 times higher (Fig. 3b). Treatment with polyphenols did not modify this increase in the initial measurements. However, there was a statistically significant decrease in the insulin values obtained in mice treated with the high dose of kaempferol between 9 and 15 months (see red arrows, p<0.05 at 9 months, p<0.005 at 15 months). Resveratrol and the low dose of kaempferol also produced statistically significant decreases in insulin levels at 15 months of treatment (p<0.05 and p<0.005, respectively). Thus, as in the case of the glucose levels, a reduction in insulin levels was only apparent after about 1 year of treatment with the polyphenols.

### Cholesterol

**Figure 4:**
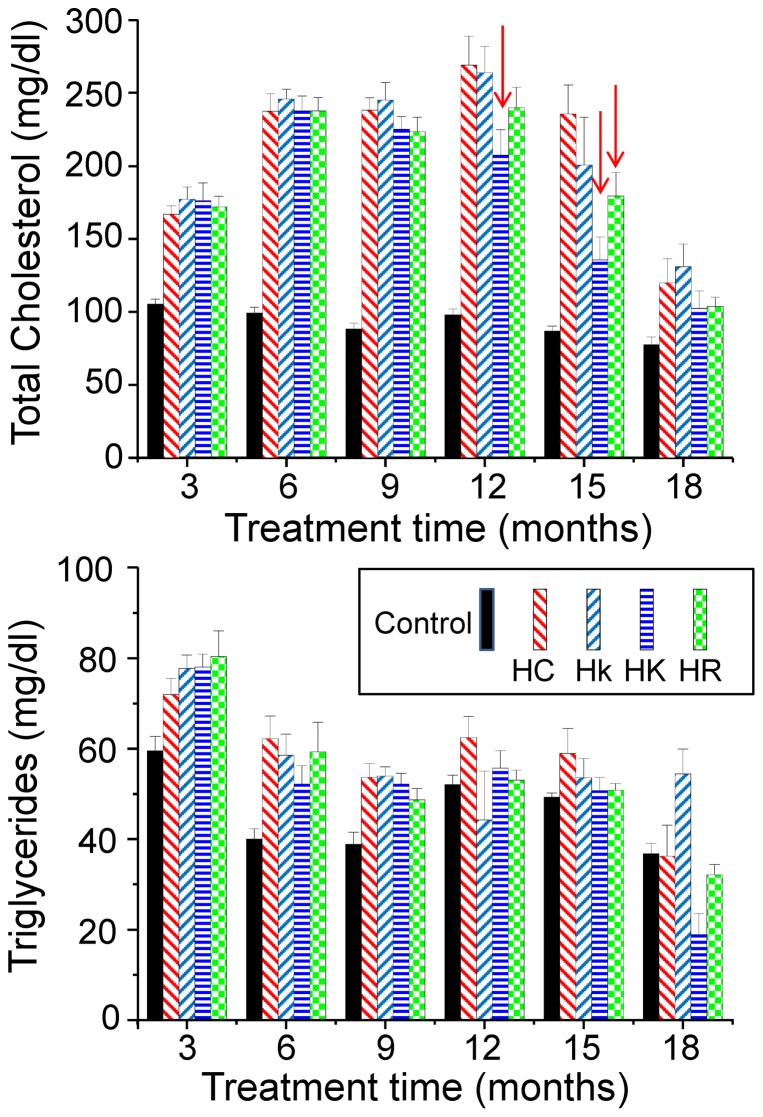
Mean blood total cholesterol (panel a) and triglycerides (panel b) values obtained every 3 months along the study, from the start of the hypercaloric diet. Arrows indicate the presence of differences statistically significant when the HC condition is compared to the HK and HR ones (see the text). Data are mean±s.e, n = 10 data for each value.

Cholesterol levels in blood were maintained at around 80–100 mg/dl in control mice throughout the study. The administration of HC diet increased these values to levels around 200 mg/dl, which were insensitive to treatment with polyphenols during the first year of treatment (Fig. 4a). In the second year there was a decrease in cholesterol levels in all the conditions. However, this decrease occurred earlier in mice treated with polyphenols. Thus, we observed a significant decrease in cholesterol levels at 15 months of treatment in mice treated with the high dose of kaempferol (p<0.005) or resveratrol (p<0.05) with respect to HC mice. Then, after 18 months of treatment, cholesterol levels were also reduced in the HC mice and the differences disappeared. In the case of mice treated with the high dose of kaempferol, there was also a significant decrease in cholesterol values (p<0.05) at 12 months of treatment. In summary, treatment with polyphenols produced little effects on cholesterol levels in the first year but accelerated the reduction of its levels associated with senescence.

### Triglycerides

Triglyceride levels in control mice stayed around 40–60 mg/dL throughout the entire study. Mice fed HC diet showed an increase of these values of about 25–30%, which was homogeneous in mice treated or not with polyphenols (Fig. 4b). This increase was maintained during the first year of treatment and thereafter the difference became smaller and tended to disappear. In any case, there were no significant differences between the data obtained in mice treated with HC diet with or without polyphenols.

### Alanine aminotransferase

**Figure 5:**
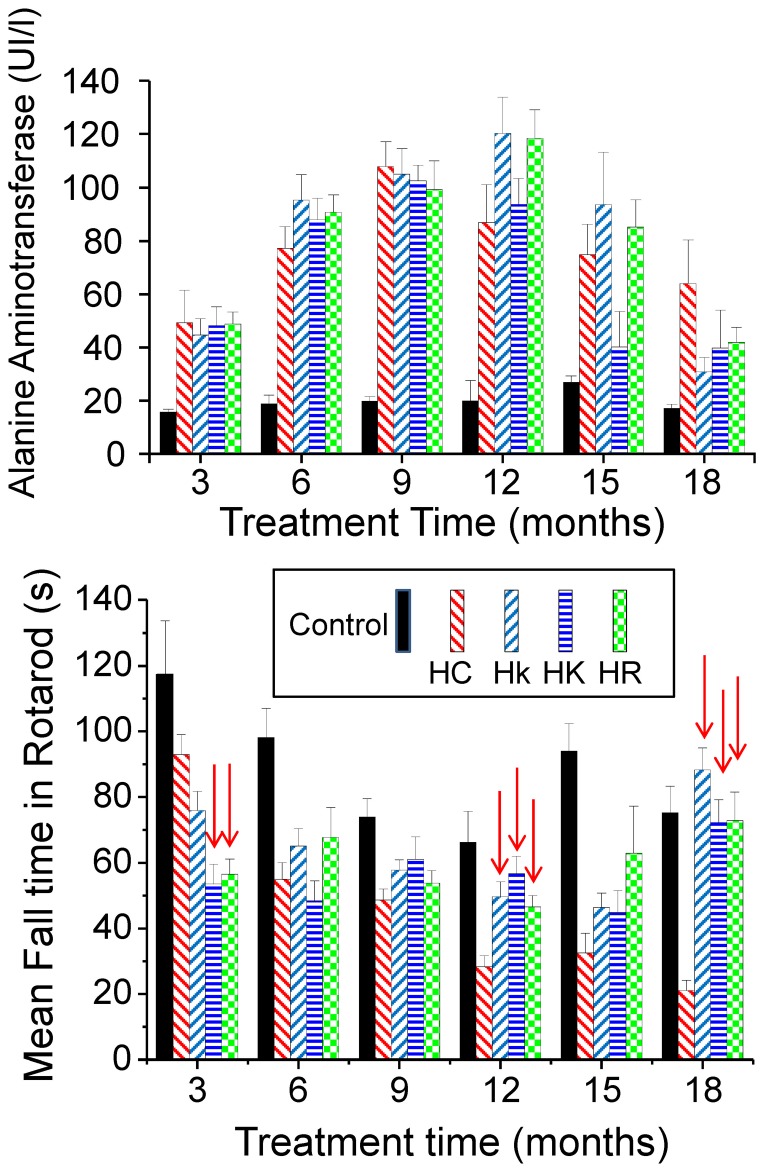
Mean blood alanine aminotransferase (panel a) and rotarod fall time (panel b) values obtained every 3 months along the study. Arrows indicate the presence of differences statistically significant when the HC condition is compared to the Hk, HK and HR ones (see the text). Data are mean±s.e, n = 10 data for each value.

Alanine aminotransferase values in blood maintained at around 20–30 IU/L throughout the entire study in control mice. In mice treated with HC diet, there was a progressive increase in these values that reached a maximum of around 100 IU/L after 9 months of treatment (Fig. 5a). Again here, there were no significant differences between the data obtained in mice treated only with HC diet and in those that had also received polyphenols.

### Motor coordination study with rotarod

In these studies, the time before fall of the mice from a rotating tube is a measure of the degree of coordination. In control mice, the time before fall decreased slightly with age, but remained between 80 and 120 seconds on average throughout the entire study. In mice treated with HC diet, there was a progressive decrease in that time, reaching values of only 30 seconds after a year of treatment (Fig. 5b). The effect of polyphenols was here quite surprising. In the first measurement, obtained after 3 months of treatment, mice treated with HC diet with kaempferol or resveratrol performed significantly worse (shorter times, p<0.005) than those treated only with diet HC. This effect disappeared at 6 and 9 months, because the values obtained for all mice with high-fat diet were here similar. Interestingly, after 12 months, data obtained by the HC diet treated mice continued to worsen, whereas those treated also with polyphenols remained stable or even improved. In fact, at 12 and 18 months, all the mice groups fed with diets containing polyphenols obtained significantly better marks (p<0.001) than those fed with HC diet.

### Histological analysis

**Figure 6:**
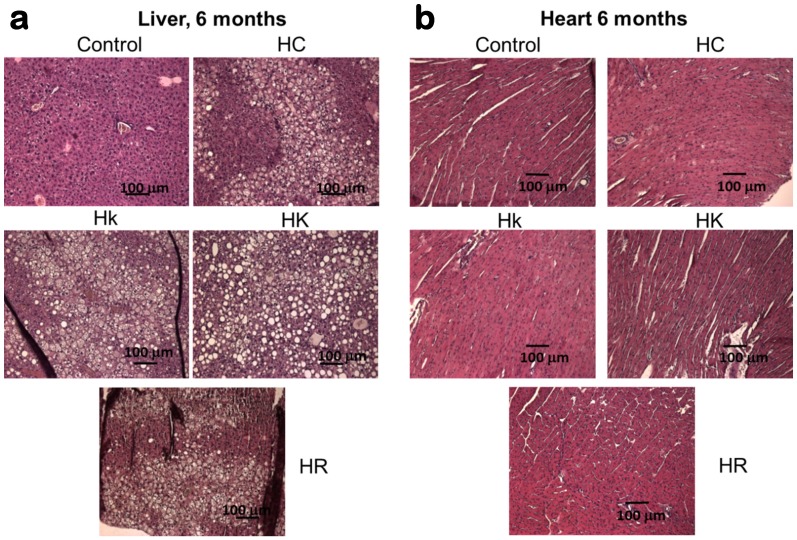
Haematoxylin and Eosin stained sections of liver (panel a) and heart (panel b), obtained from the five mice groups 6 months after the start of the hypercaloric diets and imaged in a Nikon Eclipse 80i upright microscope at 20×.

Histological studies of liver and heart samples were made at 6 and 12 months after the start of the hypercaloric diet. Fig. 6 shows typical Haematoxyline and Eosin stained sections from livers and hearts obtained 6 months after the start of the hypercaloric diet. Images obtained after 12 months provided similar results. The liver stained sections (Fig. 6a) show that all the mice under hypercaloric diet had clearly enlarged hepatocytes and a large degree of steatosis represented by the vacuolation of hepatocytes. However, after careful examination of a series of sections of each type we did not find any significant difference among the samples from mice fed only with plain hypercaloric diet and those from mice fed with hypercaloric diet plus any of the polyphenolic compounds. Regarding the heart stained sections (Fig. 6b), the hypercaloric diet induced little changes with respect to the controls, and they were not modified either by the presence of the polyphenols.

### Gene expression

We have conducted an analysis of whole genome expression arrays in the 5 groups of mice after 1 year of treatment with the different diets. Treatment with HC diet produced a significant change in the pattern of gene expression of multiple genes with metabolic importance, as has been previously described. The list of genes showing a larger change, using a 10% false positives, is in the Table S3.

Concerning the effect of polyphenols, and this is a significant discrepancy with previous data [14], the group of genes that were overexpressed or silenced was very similar in the group treated with hypercaloric diet alone and in the group fed with hypercaloric diet containing resveratrol. In fact, a direct comparison of the gene expression pattern obtained in both mice groups yielded no gene expression change between the two conditions (even using a maximum of 15% false positives).

Regarding the group fed with hypercaloric diet plus low dose of kaempferol, it was also not possible to find any gene changing expression when it was compared with the group fed only with hypercaloric diet. Similarly, in the case of the group fed with the high dose of kaempferol, just a slightly reduced expression of 6 genes by 20–30% was obtained, and only when the percentage of false positives was increased to 15% (see table S4). Some of them are mitochondrial proteins, so it may be interesting to study if they play any role in mitochondrial Ca^2+^ dynamics. However, the main conclusion of the gene expression studies is that the polyphenols added to the HC diet did not induce significant changes in the gene expression pattern generated by the HC diet.

## Discussion

Our study has sought to shed light on the controversy over the role of polyphenols on health and survival in mice. We have used resveratrol, a potent activator of sirtuins [15], and kaempferol, which is a poor activator of sirtuins [15] but a potent antioxidant with strong effects on mitochondrial metabolism activation [25]–[27]. Our macro-study covers the entire lifetime of the mice (24 months) and includes a very high number of C57BL wild-type mice, 50 mice for each condition. This makes the results highly reliable.

Our data show that the main effect of mice treatment with hypercaloric diet containing polyphenols is an initial weight loss of 5–7%, when kaempferol or resveratrol are used at a dose of 25 mg/kg body weight per day. This decrease, which requires using the high dose of kaempferol, can be seen clearly from the beginning of the treatment and is maintained throughout the study. Food ingestion during the first year was similar or higher in the drug-treated groups than in the HC control, so that the weight loss cannot be attributed to a decreased food intake. The decrease in weight induced by resveratrol in hypercaloric diet-fed mice has been described previously [14]. However, the fact that the same effect is obtained with kaempferol is important, because this flavonoid has been shown to be a poor activator of sirtuins [15]. Its effects could rather be attributed to metabolic effects such as increase in oxygen consumption or thyroid hormones synthesis [25]–[27].

Regarding the weight loss associated with senescence, it occurred 3–4 months earlier in mice treated with polyphenols compared to controls with HC diet, even with the low dose of kaempferol. This decrease was not associated with increased toxicity or mortality because the survival curves of mice treated with HC diet with or without polyphenols were similar. It could be due in part to the 5–9% reduction in food intake observed in the senescence period in all the groups treated with either kaempferol or resveratrol.

As for the study of motor coordination in rotarod, the effects of polyphenols were variable in time: negative after 3 months of treatment, indifferent between 6 and 9 months and finally positive at longer times. Notably, however, the low doses of kaempferol increased rotarod times in the last months as much as the high doses, but hardly produced any alteration at short times. The improvement in rotarod performance after long times of treatment with resveratrol has been reported before [14]. Our data here show that the same findings were also obtained with kaempferol, suggesting again that mechanisms additional to sirtuin activation may be involved.

Regarding survival, our data do not confirm the previous report that resveratrol prolongs survival of mice fed with HC diet and makes it similar to that of control mice [14]. Instead, we find that survival in the groups of mice treated with HC diet and resveratrol or kaempferol was similar to that of the group fed HC diet only.

As for the biochemical parameters, it has been reported that treatment with HC diet produces a 30% increase in glucose levels in blood and a four times increase in blood insulin levels, and that both changes were reversed in the presence of resveratrol [14]. Our data, which track more completely the glucose levels at different times of treatment, show instead that glucose levels were doubled by the HC diet and that resveratrol and kaempferol treatments were only able to reduce in part that increase during senescence, after at least 1 year of treatment. As for the insulin levels, our data show a much higher increase, between 10 and 15 times the control values, in the groups treated with HC diet. Again in this case, the polyphenols were only able to partially reverse this effect after 1 year of treatment. The origin of these partial and very delayed effects is unclear, but it could be related to the reduction in food intake and faster decrease in weight we observe during senescence in the drug-treated groups. The lack of metabolic effects of resveratrol is consistent with recent data obtained in obese men [35].

Regarding gene expression, our data show that mice fed with hypercaloric diet show a large number of changes in gene expression, confirming data previously reported by other authors [36], [37]. However, the presence of polyphenols in the hypercaloric diet hardly induced any change in gene expression with respect to the mice fed only with hypercaloric diet. These results are again contradictory with previous reports [14], which showed large changes in gene expression induced by resveratrol.

## Conclusions

In summary, we have performed here a systematic macro-study of the effect of the polyphenols resveratrol and kaempferol included in a hypercaloric diet in groups of 50 mice for each condition, monitoring weight, survival and biochemical, neurological, histological and genetic parameters along the whole life of the mice. Our results indicate that these compounds reduced mice weight by 5–7% and prolonged treatments partially reversed some of the biochemical changes induced by the hypercaloric diet. However, the polyphenols did not significant modify survival or liver gene expression.

## Supporting Information

Table S1 Food ingestion in the different groups measured in both g⋅mice^−1^⋅day^−1^ or kcal⋅mice^−1^⋅day^−1^.
(DOCX)

Table S2 Significance of mice weight differences between drug treated (Hk, HK and HR) and hypercaloric control HC (*, p<0.05; **, p<0.005; ***, p<0.001).
(DOCX)

Table S3 Differential gene expression obtained by comparing gene expression arrays for HC mice versus controls.
(XLSX)

Table S4 Differential gene expression obtained by comparing gene expression arrays for HK versus HC mice.
(XLSX)
