# Supplementary material for: Effects of Long-Term Feeding of the Polyphenols Resveratrol and Kaempferol in Obese Mice
Source: PLoS One. 2014 Nov 11;9(11):e112825. doi: 10.1371/journal.pone.0112825 (PMC4227868; doi:10.1371/journal.pone.0112825)
Supplement: Table S1 — Food ingestion in the different groups measured in both g⋅mice−1⋅day−1 or kcal⋅mice−1⋅day−1. (DOCX) [file pone.0112825.s001.docx]

**Supplementary Table S1**

**Table S1.** Food ingestion in the different groups measured in both g•mice^-1^•day^-1^ or kcal•mice^-1^•day^-1^.

|  | **Whole period (n=197)** | **1-12 months**  **(n=112)** | **13-21 months**  **(n=85)** |
| --- | --- | --- | --- |
| **Control** | 2.68±0.02 g (10.32±0.07 kcal) | 2.73±0.02  (10.51±0.07 kcal) | 2.62±0.02  (10.09±0.07 kcal) |
| **HC** | 2.64±0.02  (13.83±0.10 kcal) | 2.63±0.02  (13.78±0.10 kcal) | 2.67±0.02  (14.00±0.10 kcal) |
| **Hk** | 2.55±0.02  (13.36±0.10 kcal) | 2.63±0.02  (13.78±0.10 kcal) | 2.44±0.02  (12.79±0.10 kcal) |
| **HK** | 2.52±0.02  (13.20±0.10 kcal) | 2.58±0.02  (13.52±0.10 kcal) | 2.44±0.03  (12.79±0.15 kcal) |
| **HR** | 2.63±0.02  (13.78±0.10 kcal) | 2.70±0.02  (14.15±0.10 kcal) | 2.53±0.04  (13.26±0.20 kcal) |
