# Supplementary material for: Effects of Long-Term Feeding of the Polyphenols Resveratrol and Kaempferol in Obese Mice
Source: PLoS One. 2014 Nov 11;9(11):e112825. doi: 10.1371/journal.pone.0112825 (PMC4227868; doi:10.1371/journal.pone.0112825)
Supplement: Table S2 — Significance of mice weight differences between drug treated (Hk, HK and HR) and hypercaloric control HC (*, p<0.05; **, p<0.005; ***, p<0.001). (DOCX) [file pone.0112825.s002.docx]

**Supplementary Table S2**

**Table S2.** Significance of mice weight differences between drug treated (Hk, HK and HR) and hypercaloric control HC (*, p<0.05; **, p<0.005; ***, p<0.001).

| Treatment time (months) | Significance vs HC | | |
| --- | --- | --- | --- |
|  | Hk | HK | HR |
| 0.5 |  | * | *** |
| 1 |  | ** | *** |
| 1.5 |  | *** | *** |
| 2 |  | *** | *** |
| 2.5 |  | * | *** |
| 3 |  |  | ** |
| 3.5 |  |  | * |
| 4.5 |  |  | * |
| 5 |  |  | ** |
| 5.5 |  |  | ** |
| 6 |  |  | * |
| 6.5 |  | * | ** |
| 7 |  | ** | *** |
| 7.5 |  |  | * |
| 8 |  | *** | *** |
| 8.5 | * | *** | *** |
| 9 | * | *** | *** |
| 9.5 |  | * | ** |
| 10 | ** | *** | *** |
| 10.5 |  | *** | ** |
| 11 | * | *** | *** |
| 11.5 | * | *** | ** |
| 12 | * | *** | * |
| 12.5 | * | ** | * |
| 13 | * | *** |  |
| 13.5 | * | *** |  |
| 14 | * | *** |  |
| 14.5 | ** | *** |  |
| 15 | ** | *** | * |
| 15.5 | ** | *** | * |
| 16 | * | *** | * |
| 16.5 | * | *** | * |
| 17 | ** | *** | * |
| 17.5 | * | *** | * |
| 18 | * | *** | * |
| 18.5 | * | * | * |
| 19 | * |  |  |
| 19.5 | * |  |  |
| 20 |  |  |  |
